# Electrocardiographic diagnosis of atrial cardiomyopathy to predict atrial contractile dysfunction, thrombogenesis and adverse cardiovascular outcomes

**DOI:** 10.1038/s41598-021-04535-7

**Published:** 2022-01-12

**Authors:** Björn Müller-Edenborn, Jan Minners, Cornelius Keyl, Martin Eichenlaub, Nikolaus Jander, Sherif Abdelrazek, Christoph Ahlgrim, Jürgen Allgeier, Heiko Lehrmann, Franz-Josef Neumann, Thomas Arentz, Amir Jadidi

**Affiliations:** 1 Department of Cardiology and Angiology II, Heart Center, Section of Electrophysiology, University of Freiburg, Südring 15, 79189 Bad Krozingen, Germany; 2 Department of Cardiology and Angiology II, Heart Center, Section of Anesthesiology, University of Freiburg, Bad Krozingen, Germany; 3 Department of Cardiology and Angiology II, Heart Center, University of Freiburg, Bad Krozingen, Germany

**Keywords:** Cardiology, Neurology

## Abstract

Thromboembolism and stroke are dreaded complications in atrial fibrillation (AF). Established risk stratification models identify susceptible patients, but their discriminative properties are poor. Atrial cardiomyopathy (ACM) is associated to thromboembolism and stroke in smaller studies, but the modalities used for ACM-diagnosis (MRI and endocardial mapping) are unsuitable for widespread population screening. We aimed to investigate an ECG-based diagnosis of ACM using amplified p-wave analysis (APWA) for stratification of thromboembolic risk and cardiovascular outcome. In this case–control study, ACM-staging was performed using APWA on digital 12-lead sinus rhythm-ECGs in patients with LAA-thrombus and a propensity-score-matched control-cohort. Left atrial contractile function and thrombi were evaluated by transesophageal echocardiography (TEE). Outcome for MACCE including death was assessed using official registries and structured phone interviews. Left-atrial appendage [LAA]-thrombi and appropriate sinus rhythm-ECGs for ACM-staging were found in 109 of 4086 patients that were matched 1:1 to control patients without thrombus (218 patients in total). Both cohorts were comparable regarding cardiovascular risk factors, anticoagulants and CHA2DS2-VASC-score. ACM-stages 1 to 3 (equivalent to no, moderate and extensive ACM) were found in 63 (57.8%), 36 (33.0%) and 10 (9.2%) of patients without and 3 (2.8%), 23 (21.1%) and 83 (76.1%) of patients with LAA-thrombi. Atrial contractile function decreased from ACM-stages 1 to 3 (LAA-flow velocities 38 ± 16 cm/s, 31 ± 15 cm/s and 21 ± 12 cm/s; p < 0.0001), while the likelihood for LAA-thrombus increased (2.8%, 21.1% and 76.1%, p < 0.001). Multivariable analysis confirmed an independent odds ratio for LAA-thrombus of 24.6 (p < 0.001) per ACM-stage. Two-year survival free of stroke/TIA, hospitalization for heart failure, myocardial infarction or all-cause death was strongly reduced in ACM-stage 3 (53.8%) compared to no or moderate ACM (82.8% and 84.7%, respectively; p < 0.0001). Electrocardiographic diagnosis of ACM identifies patients with atrial contractile dysfunction and atrial thrombi at risk for adverse cardiovascular outcomes and death.

## Introduction

More than eight million people in the European Union are affected by atrial fibrillation (AF), and its prevalence is predicted to rise further in the coming decades^1^. AF has a significant impact on quality of life, morbidity and mortality, with systemic thromboembolism and ischemic stroke being among the most dreaded complications of AF^2^.

Oral anticoagulation (OAC) significantly reduces the risk for thrombus formation in the left-atrial appendage (LAA) and subsequent stroke and thromboembolism, but major and potentially fatal bleedings are important side effects. The identification of low risk-patients, in whom the disadvantages of OAC likely outweigh the benefit, and high risk-patients, who share an important stroke risk and are therefore best suited for OAC, is challenging in clinical practice. Several clinical risk stratification models were validated for this purpose, but their predictive properties (e.g. c-statistic of 0.64 for the widely used CHA2DS2-VASC-score) are limited^3^.

In the recent past, large efforts focused on the contribution of atrial cardiomyopathy (ACM) on thrombus formation and stroke, and on identification of patients with presumed ACM^4,5^. In this context, left-atrial endocardial contact mapping and contrast-enhanced atrial MRI were demonstrated to identify ACM and relate to prior ischemic stroke, but invasiveness, prohibitive costs and low availability limit the practicability of these findings, in particular for large-scale primary-preventive screening purposes^6,7^. Nevertheless, current ESC-guidelines recognize the importance of ACM and emphasize the need for characterization of ACM in AF-patients^1^.

Our group recently demonstrated that structured analysis of a highly amplified digital 12-lead ECG (APWA, amplified p-wave-analysis) allows to identify three distinct severity grades of ACM in patients undergoing pulmonary vein isolation, with striking differences in AF ablation-outcome^8,9^. For the current study, we hypothesized that APWA may also be suitable to stratify patients with ACM to their individual risk for atrial contractile dysfunction, thrombogenesis and adverse cardiovascular outcomes.

## Methods

### Study design and study population

We performed a case–control study to evaluate the association between atrial contractile function, LAA thrombus and amplified sinus-P-wave-characteristics in digitally recorded 12-lead surface ECGs and associated risk factors in patients with atrial fibrillation scheduled for electrical cardioversion. Consecutive patients undergoing transesophageal echocardiography (TEE) with intended cardioversion at our hospital between January 2007 and December 2018 were screened for this study. TEE was indicated (a) in patients without prior oral anticoagulation, (b) in those using vitamin K-antagonists who demonstrated one or more measurements with an international normalized ratio < 2.0 in the preceding four weeks, or (c) all patients receiving novel oral anticoagulants. The indication for TEE in patients under NOAC was based on the incomplete clinical safety data up until 2018.

Patients were included when TEE was performed in AF and cardioversion intended. Exclusion criteria were insufficient image quality on TEE and unavailability of a digital 12 lead-ECG in sinus rhythm recorded within three months from TEE. The primary endpoint of the current study was LAA flow velocity measured at the index TEE in atrial fibrillation and the likelihood for LAA-thrombus according to the APWA-derived ACM-stage. Secondary endpoint was future MACCE (stroke/TIA, myocardial infarction, hospitalization for heart failure and all-cause death). For follow-up of stroke/TIA, myocardial infarction and hospitalization for heart failure, all available medical records were reviewed by independent physicians and structured phone interviews with study participants and treating physicians conducted. All-cause mortality was taken from the official German death registry. MACCE occurring during the index hospitalization were not included in the final analysis. The current study was approved by the local institutional review board of the University of Freiburg and patients gave informed consent. All methods used in the current manuscript were carried out in accordance with relevant guidelines and regulations.

### Transesophageal echocardiography and amplified P-wave-analysis

All TEE were evaluated for presence of LAA thrombus by two independent cardiologists. The left atrium and the left atrial appendage were inspected closely for the presence of thrombus. Left atrial appendage was visualized in at least two orthogonal planes carefully optimized to avoid shadowing artifacts. Flow velocity signals were obtained 1 cm below the entrance of the left atrial appendage and spectral pulsed-wave Doppler was monitored for 10 s; peak LAA flow velocity over the monitored interval was measured. Thrombus formation was diagnosed in the presence of a solid wall-adherent structure detectable in two orthogonal planes carefully differentiating them from musculi pectinati and other anatomical structures.

**Figure 1:**
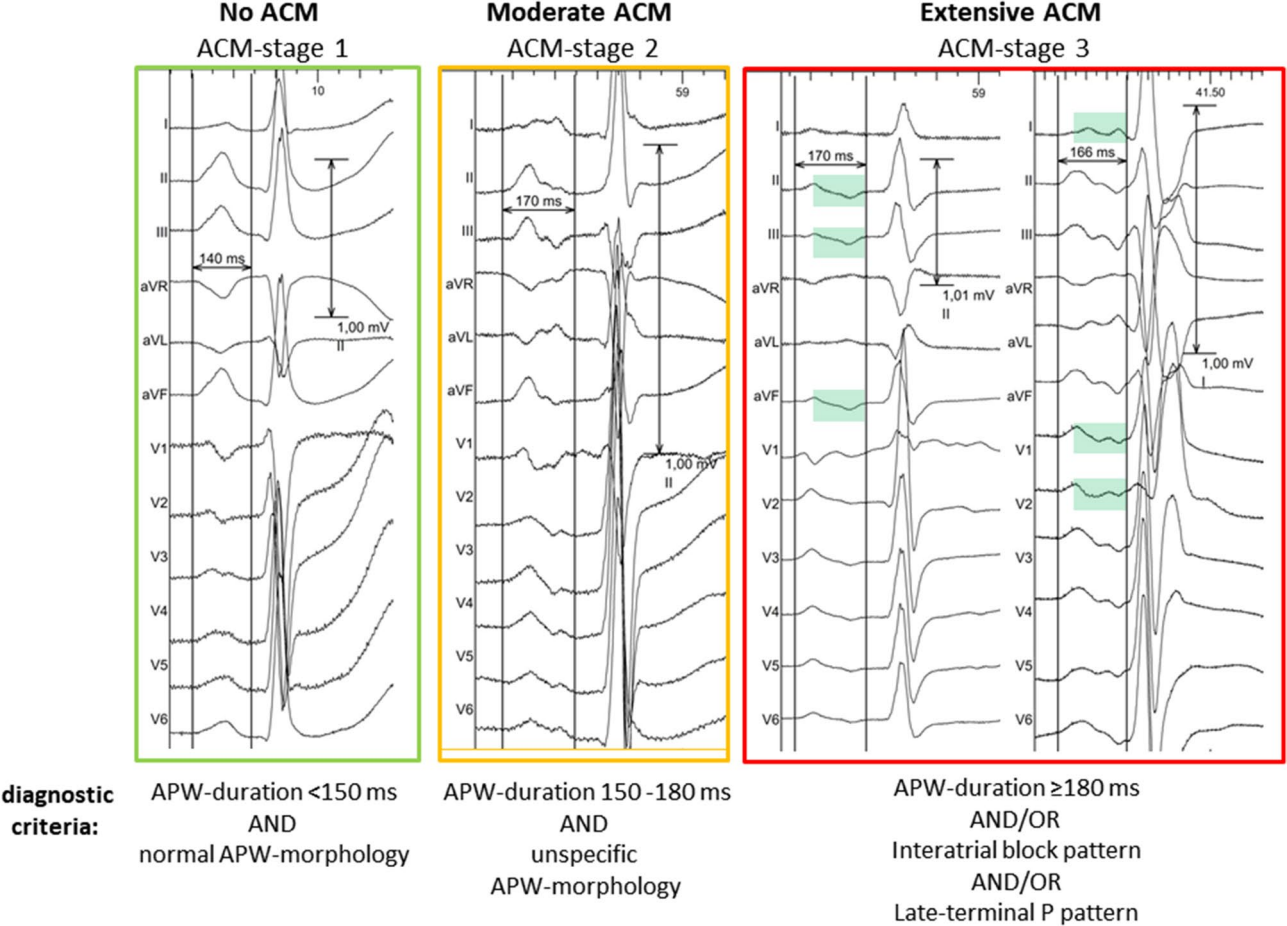
Electrocardiographic diagnosis of atrial cardiomyopathy. Twelve-lead surface ECGs from four representative patients with 100 mm/s sweep speed and amplification of 40–80 mm/mV. In ACM-stage 1 (green box, no ACM), APW-duration is < 150 ms with normal morphology. In ACM-stage 2 (yellow box, moderate ACM), APW-duration is prolonged from 150 to 180 ms with normal or unspecific morphology. ACM-stage 3 (red box, extensive ACM) is diagnosed with prolongation of APW ≥ 180 ms or when pre-specified morphological criteria (interatrial block [IAB] defined as biphasic positive–negative P-waves in 3 of 3 inferior leads II, III and aVF or late-terminal P defined as a positive deflection [reflecting activation of the left atrial appendage] in one or more lateral leads preceded by an isoelectric interval [reflecting ubiquitous low-voltage within the left atrial body]) are met. APW amplified P-wave; ACM, atrial cardiomyopathy. ECGs exported using EP-Labsystem Pro V4.0, Boston Scientific, USA.

Amplified p-wave analysis (APWA) was performed by two independent investigators who were blinded for the patient characteristics using a digitally recorded and amplified 12-lead surface ECG (AT104PC, Schiller, Switzerland) and measured using the SEMA200-software (Schiller, Switzerland) with following settings: amplification 40–80 mm/mV, sweep speed 100–200 ms). The methodology of APWA to diagnose three distinct stages of ACM was described previously in detail^8,9^. Briefly, the diagnostic algorithm is based on the duration of amplified P-wave (APW) and defined morphological criteria (interatrial block pattern or late-terminal-P pattern): an APW-duration ≤ 150 ms with unspecific morphology is considered physiological (no ACM; ACM-stage 1). An APW-duration of 150 – 180 ms with unspecific APW-morphology is found in moderate ACM (ACM-stage 2). Extensive ACM (ACM-stage 3) is present when APW-duration is importantly prolonged at > 180 ms and/or demonstrates an interatrial block- or late-terminal-P pattern. Inter-observer consistency for ACM classification was 0.91 (Intraclass-correlation coefficient, two-way mixed analysis for absolute agreement). In cases of disagreement for p-wave duration, the mean p-wave duration of the two investigators was calculated (necessary in 8/218 patients, 3.6%). See Fig. 1 and its legend for further details.

### Statistics

Statistical analysis was performed using SPSS version 25.0 for Macintosh (IBM Corporation, Armonk, New York) or GraphPad Prism version 8 for Macintosh (GraphPad Software, La Jolla, California). Normally distributed data are expressed as mean ± SD, and skewed data are expressed as median (interquartile range). Intergroup comparison between two groups was performed using student’s t-test for normally distributed data and Mann–Whitney-U-test for skewed data.

A propensity score-matched control cohort of AF patients without LAA thrombus on TEE (performed prior to the planned electrical cardioversion) served as reference for this study. The propensity score, expressing the probability of a LAA thrombus, was calculated by multivariate logistic regression with variables that were previously shown to be related with LAA thrombus as covariates (age, gender, LA diameter and left ventricular function). Matching on the logit of propensity score was performed using the R-based extension-bundle PSMATCHING3^10^. We selected the nearest neighbor method using calipers of width 0.2 of the standard deviation of the logit of the propensity score. The balance in the included covariates was assessed by calculating the standardized mean difference for each covariate. In addition, the total relative imbalance measure L1 was calculated for the original and the matched data. The histograms of the density of propensity scores for observations before and after matching with overlaid Kernel density estimate, the dot plots of the propensity scores before and after matching, and the dot plots of the standardized mean differences for all covariates before and after matching are given in Supplemental Fig. 1. Variables were compared between groups by taking the matched nature of the data into account, as suggested by Austin^11^. Variables that differed between patients with and without LAA thrombus at a significance level < 0.10 and/or which were judged to be clinically significant were analyzed by conditional logistic regression, using Cox regression analysis with “Time” as a grouping variable. For multivariate models to predict LAA-thrombi, established cardiovascular risk factors that were previously related to atrial thrombogenesis and/or stroke, were chosen as covariates. The use of oral anticoagulants was included as covariate due to their clinical importance.

Future MACCE was calculated using the Kaplan–Meier-method and groups were compared by log rank-test and cox-regression. Follow-up was censored at 720 days to preclude insufficient patient numbers. For all analyses, a two-tailed p < 0.05 was considered significant.

## Results

A total of 4862 TEE were conducted between January 2007 and December 2018 in 4086 individual patients with atrial fibrillation scheduled for electrical cardioversion. Solid LAA thrombus was found in 192 of 4086 patients (4.6%). A digitally recorded sinus rhythm 12-lead-ECG within three months from the date of TEE was available in 109 of 192 patients with LAA thrombus. The remainder of patients were either not cardioverted and had no ECG in sinus rhythm (n = 78), or the technical quality of the ECG was deemed insufficient for analysis (n = 5). The LAA-thrombus cohort was propensity score-matched to a control cohort of 109 patients without evidence of LAA-thrombus (Supplemental Fig. 2). Descriptive data of LAA-thrombus patients and matched control patients are given in Table 1. Patients with LAA thrombus did not differ from matched control patients in terms of age, sex or CHA2DS2VASC-score, as well as AF-type, prior use of oral anticoagulation or echocardiographic parameters of left-atrial or left-ventricular structure and function. ACM-stages 1 to 3 (no/moderate/extensive ACM) were found in 68 (35.4%), 57 (29.7%) and 93 (42.7%) patients, respectively.

**Table 1:** Patient characteristics.

Variable	Control n = 109	LAA thrombus n = 109	P value
Male sex	41 (37.6)	35 (32.1)	0.394
Age (y)	67.5 (10.2)	69.8 (10.2)	0.096
CHADSVASC	3.24 (1.63)	3.2 (1.46)	0.860
**AF-type**			0.661
Paroxysmal	7 (6.4)	2 (1.8)	
Persistent	69 (63.3)	79 (72.5)	
Long-persistent	9 (8.3)	4 (3.7)	
Unknown	23 (21.1)	24 (22)	
**AF-duration**			0.869
< 6 months	77 (70.4)	74 (67.9)	
6 to > 12 months	9 (8.3)	11 (10.1)	
Unknown	23 (21.3)	24 (22.0)	
**Antiarrhhythmic drugs**			0.598
None	26 (23.9)	19 (17.49)	
Class 1c	2 (1.8)	4 (3.7)	
Class 2	42 (38.5)	49 (45.0)	
Class 3	36 (33.0)	32 (29.4)	
**Prior oral anticoagulants**			0.879
None	19 (18.8)	21 (19.3)	
Vitamin K-antagonists	46 (45.5)	52 (47.7)	
NOAC	33 (32.6)	33 (30.4)	
LMWH	3 (3.0)	3 (2.8)	
Prior antiplatelets	31 (31)	31 (29.6)	0.904
Hypertension	84 (77.8)	88 (80.7)	0.838
Diabetes	21 (19.4)	18 (16.5)	0.369
History of stroke or TIA	10 (9.2)	11 (10.1)	0.853
Vascular disease	34 (31.5)	47 (43.1)	0.069
LA diameter (mm)	48 (5)	49 (5)	0.125
LA volume (ml)	112.3 (37.3)	120.0 (39.9)	0.146
LA indexed volume (ml/sqm)	28.2 (10.9)	31.1 (11.4)	0.122
LV end-diastolic diameter (mm)	56 (9)	56 (9)	0.786
LV ejection fraction (%)	37.5 (12)	35.1 (13)	0.164
LV systolic dysfunction n (%)	82 (76.6)	86 (78.9)	0.689
**Amplified ECG-parameters**			
APW-duration	149 (16)	183 (30)	< 0.0001
interatrial block pattern	7 (6.4)	25 (22.9)	< 0.0001
late-terminal-P pattern	2 (1.8)	31 (28.4)	< 0.0001
**Predicted ACM-stage**			< 0.0001
No ACM	65 (57.8)	3 (2.8)	
Moderate ACM	34 (33.0)	23 (21.1)	
Extensive ACM	10 (9.2)	83 (76.1)	

Values are given as n (%) or mean and standard deviation.

*ACM* atrial cardiomyopathy, *LA* left atrium, *LMWH* low molecular weight heparin, *LV* left ventricle, *TIA* transient ischemic attack.

### Amplified P-wave-analysis-derived atrial cardiomyopathy-stage is associated with left atrial contractile function

**Figure 2:**
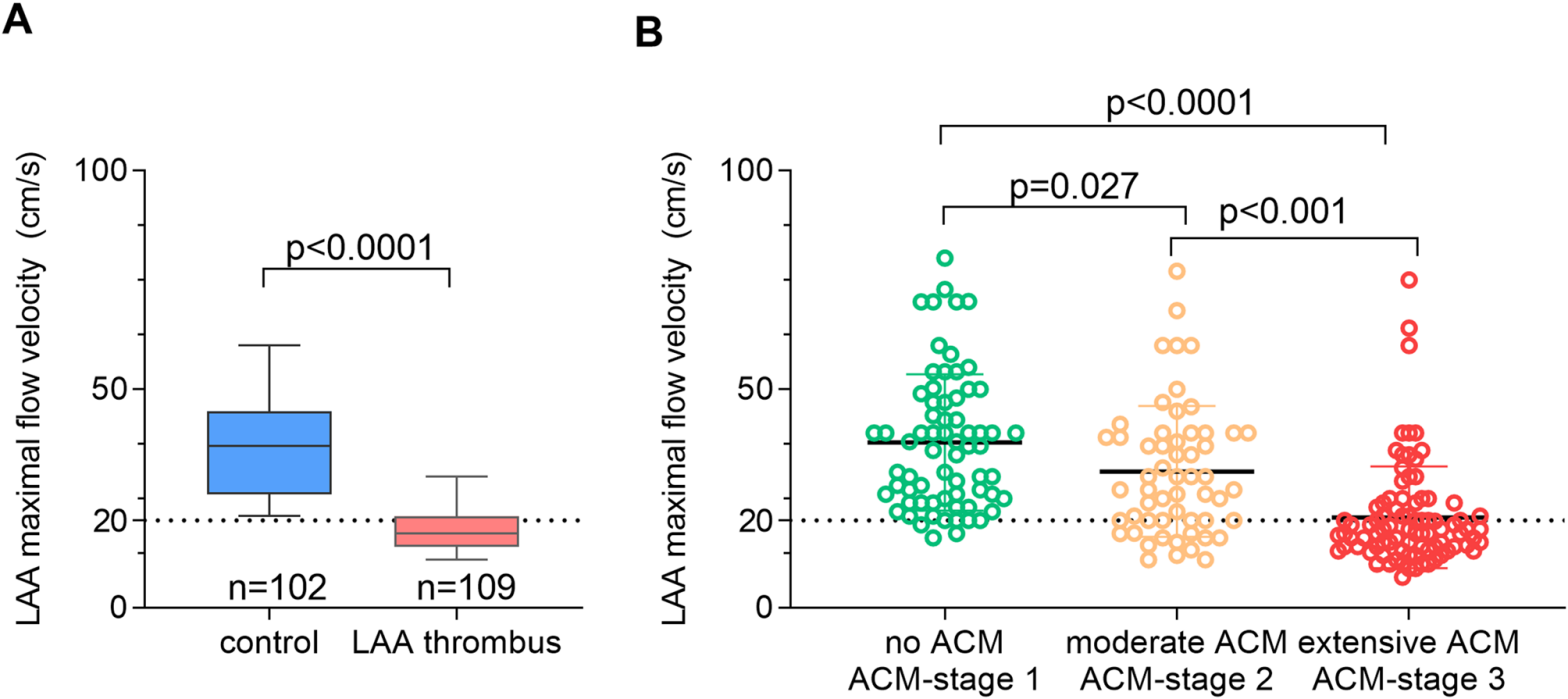
Amplified P-wave-analysis relates to left atrial contractile function. Flow velocities in the left atrial appendage (LAA) in patients with LAA thrombus (in red) as compared to propensity-matched control patients (in blue). The dashed line indicates a flow velocity of 20 cm/s and lower that was previously shown to relate to overt stroke (**A)**. LAA-flow velocities decrease from healthy patients with ACM-stage 1 (no ACM, green) to patients with ACM-stage 2 (moderate ACM, orange) and ACM-stage 3 (extensive ACM, red, **B)**. ACM atrial cardiomyopathy; LAA, left atrial appendage.

Impaired flow velocities in the LAA ≤ 20 cm/s were previously demonstrated to promote thrombus formation in the LAA and are associated with an increased risk of cardioembolic stroke^12^. In the current study, LAA flow velocities were significantly lower in patients with solid LAA thrombus (19 ± 10 cm/s vs. 38 ± 15 cm/s in controls, p < 0.0001; Fig. 2A). LAA-slow flow ≤ 20 cm/s was associated with prolonged APW-duration (Supplemental Fig. 3A, 184 ± 31 ms vs. 156 ± 22 ms, p < 0.0001) as well as presence of pathologic APW-morphologies (Supplemental Fig. 3B; p < 0.0001 for interatrial block-pattern, p < 0.0001 for late-terminal-P pattern). Accordingly, LAA-flow velocities decreased continuously from APWA-derived ACM-stage 1 (no ACM) to ACM-stage 3 (extensive ACM; Fig. 2B; 38 ± 16 cm/s in ACM-stage 1, 31 ± 15 cm/s in ACM-stage 2 and 21 ± 12 cm/s in ACM-stage 3; p < 0.0001). Only 10.3% of patients without ACM (ACM-stage 1) fell below the critical threshold for thrombus formation of ≤ 20 cm/s, whereas this was the case in 32.1% and 67.8% of patients with moderate and extensive ACM (ACM-stages 2 and 3, respectively; p < 0.0001). The strong association of ACM-stage and LAA-slow flow persisted also when adjusting for common cardiovascular risk factors and mitral regurgitation (OR 6.841 per unit increase in ACM-stage [95% CI 3.40–13.75; p < 0.0001]; Supplemental Table 1). In eight patients (3.6%), LAA flow velocities were not documented.

### Amplified P-wave analysis identifies patients with left atrial thrombus

**Figure 3:**
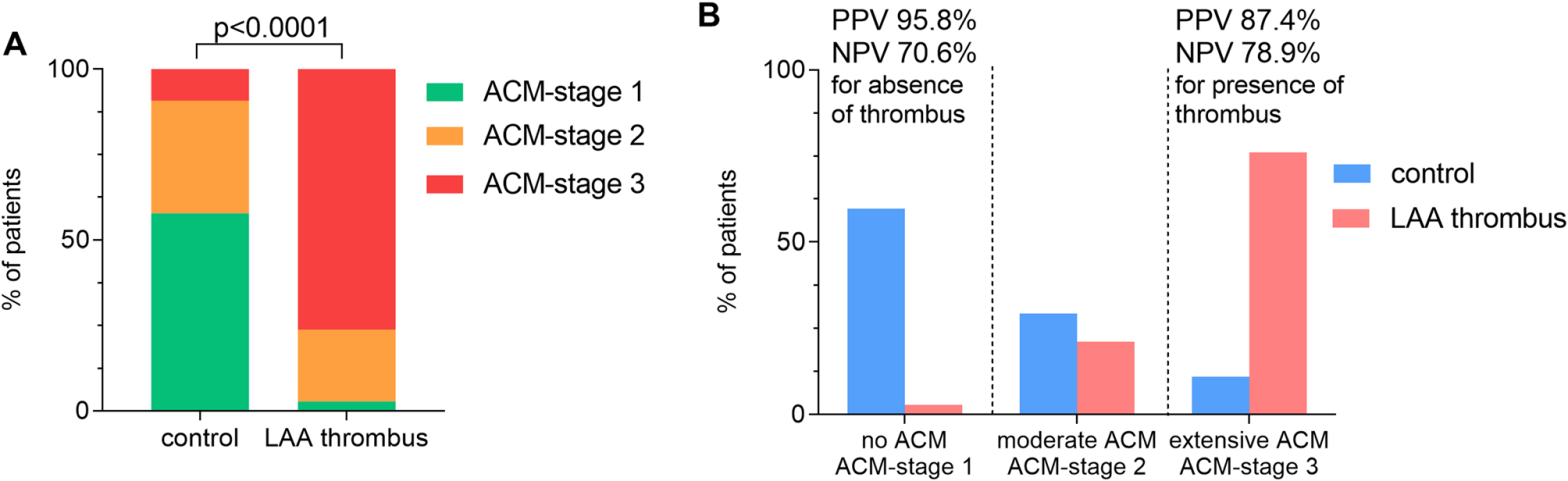
Amplified P-wave analysis identifies patients with left atrial thrombus. Overview of ACM-stages (ACM-stage 1 in green, ACM-stage 2 in orange, ACM-stage 3 in red) in patients with LAA-thrombus and matched control patients (**A**). APWA-derived ACM stages according to the presence or absence of LAA-thrombus are shown in **(B)**. *APWA* amplified P-wave analysis, *ACM* atrial cardiomyopathy.

Patients with solid LAA thrombus had longer APW-duration (Supplemental Fig. 4A; 184 ± 30 ms vs. 150 ± 16 ms, p < 0.0001) and were more likely to demonstrate pathologic APW-morphologies (Supplemental Fig. 4B; p < 0.0001 for interatrial block-pattern, p < 0.0001 for late-terminal-P pattern) compared to matched control patients without thrombus. Moderate or extensive ACM (ACM-stages 2 and 3) was seen in 106/109 patients with LAA thrombus (97.2%), as compared to 44/109 patients (40.4%) without LAA thrombus (Fig. 3A, p < 0.001). In particular, electrocardiographic diagnosis of “no ACM” (ACM-stage 1; short APW-duration < 150 ms and normal morphology) and “extensive ACM” (ACM-stage 3; prolonged APW-duration ≥ 180 ms and/or pathologic morphological APW-patterns) had good discriminative power for absence or presence of LAA-thrombus, respectively (Fig. 3B; PPV 95.8%/NPV 70.6% for absence of LAA-thrombus in ACM-stage 1, PPV 87.4%/NPV 78.9% for presence of LAA-thrombus in ACM-stage 3). APWA-derived ACM-stages were also found to be significant independent predictors of LAA thrombus (estimated OR 24.594, 95% CI 5.66–106.83, p < 0.001) in a multivariable model that included established cardiovascular risk factors and the use of OAC (Table 2).

**Table 2:** Logistic regression analyses of predictors for left-atrial thrombus.

	Univariate analyses	Multivariate analyses		
Variable	P value	OR	95% CI	P value
Age	0.117	0.972	0.92–1.03	0.345
male sex	0.367	0.339	0.05–2.18	0.254
diabetes	0.371	1.723	0.35–8.52	0.504
LV systolic dysfunction	0.655	8.272	0.51–133.1	0.136
history of stroke or TIA	0.827	0.744	0.13–4.40	0.744
vascular disease	0.072	0.903	0.25–3.21	0.875
hypertension	0.842	0.508	0.08–3.20	0.471
prior oral anticoagulants	0.695	1.040	0.25–4.40	0.958
APWA-derived ACM-stage (per increase in stage)	< 0.0001	24.594	5.66–106.83	< 0.001

*APWA* amplified p-wave analysis, *ACM* atrial cardiomyopathy, *LV* left ventricular, *TIA* transient ischemic attack.

### Future MACCE and electrocardiographic evidence of atrial cardiomyopathy

**Figure 4:**
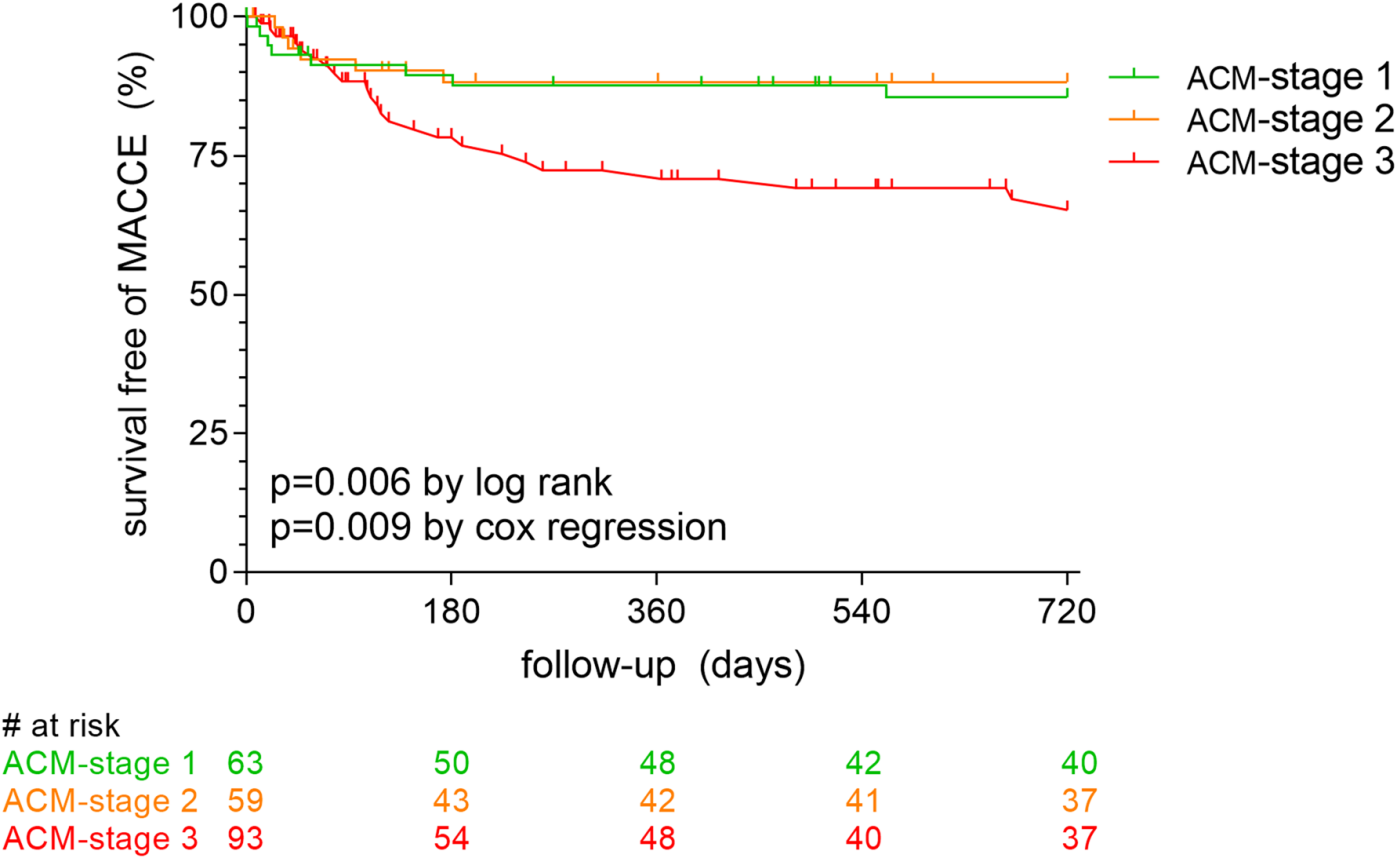
Future MACCE and electrocardiographic evidence of ACM. Kaplan–Meier-curves for survival free of MACCE (stroke or transient ischemic attack, myocardial infarction, hospitalization for heart failure or all-cause death) in patients with ACM-stages 1 (no ACM, in green), 2 (moderate ACM, in orange) or 3 (extensive ACM, in red). ACM atrial cardiomyopathy.

Sixty-three patients (28.9%) experienced MACCE (stroke/TIA, myocardial infarction, hospitalization for heart failure or all-cause death) during a mean follow-up of 469 ± 295 days. The rate of MACCE was primarily driven by all-cause death (n = 33), followed by hospitalization for heart failure (n = 32) and stroke/TIA (n = 7). None of the patients experienced myocardial infarction. Patients with extensive ACM (ACM-stage 3) were threefold more likely to experience a MACCE (Fig. 4; 46.2%, HR 3.267, 95% CI 1.68–6.35, p < 0.0001) compared to ACM-stages 1 and 2 (16.7% and 15.3%, respectively). These differences persisted when adjusting the analysis to patient age (HR 2.883, 95% CI 1.46–5.70, p = 0.002) and when adjusted for common cardiovascular risk factors (Supplemental Fig. 5). No significant differences were noted between patients of ACM-stages 1 and 2 (p = 0.802 unadjusted for age and p = 0.816 adjusted for age). Mortality data was unavailable in five patients (2.3%).

## Discussion

We report three main findings: First, electrocardiographic diagnosis of atrial cardiomyopathy (ACM) identifies three distinct severity grades of ACM in patients who are otherwise homogeneous with regard to established cardiovascular risk factors such as those incorporated in the CHA2DS2VASC-score. Second, ACM-severity is associated with atrial contractile dysfunction and presence of left-atrial thrombi, both established risk factors for overt stroke. And third, severe ACM is related to adverse cardiovascular outcomes and death.

### The role of atrial cardiomyopathy in thrombogenesis and stroke

Several recent trials highlight the importance of ACM for atrial thrombogenesis and stroke: Müller et al. reported that ACM (as diagnosed by endocardial contact mapping of the LA) related to prior stroke and silent cerebral ischemia^6^. Akoum and Daccarett et al. used delayed-enhanced atrial MRI (DE-MRI) to diagnose ACM, and equally found that ACM was associated with LAA thrombi and history of stroke^13,14^. In line with these studies and others, the current ESC-guidelines on AF recognize the importance of ACM, and recommend screening for ACM to risk-stratify patients with AF^1^.

The widespread use of endocardial contact mapping and DE-MRI for this purpose is however importantly limited due to peri-procedural risks, prohibitive costs, and low availability. In light of these restrictions and to answer the need for large-scale screening for ACM in primary prevention, several population-based studies evaluated P-wave-characteristics. These studies were based on the assumption that an atrial cardiomyopathy might equally affect the electrical atrial function and hence P-wave-characteristics as well as the thromboembolic risk^15^. The best-studied P-wave-parameters in this context are P-terminal force in the precordial lead V1 (PTFV1), prolongation of the standard (non-amplified) P-wave, and P-wave area.

It is important to note that these parameters are thought to reflect left atrial electrical abnormalities, but none of them was actually validated against invasive electro-anatomical maps of the LA^16–18^. In addition, the use of non-digital ECG source data in combination with standard amplifications (10 mm/mV) and sweep speeds (25 mm/s) underestimates the true left atrial activation time that is incorporated as the time-domain in all of the above-mentioned parameters. This is particularly true in patients with extensive ACM who have widespread left atrial low-voltage areas at endocardial mapping and low-amplitude p-waves in the surface-ECG^8,9^, leading to underdetection of the non-amplified P-wave-duration and -morphology.

### Amplified P-wave analysis for diagnosis of atrial cardiomyopathy

The current study uses amplified P-wave-analysis (APWA) on digital 12-lead-ECGs to determine the characteristics of the P-wave in sinus rhythm^8,9^. In contrast to the previously reported ECG-parameters, APWA is based on digital 12-lead-ECGs recorded with high quality/low noise in the absence of additional notch filtering or other noise-cancelling filters. The digital raw data are then amplified at post-processing to ensure that low-amplitude signals marking the end of the P-wave are adequately visualized particularly in patients with advanced ACM. This method was validated against high-density electro-anatomical activation- and voltage-maps of the LA and demonstrates a high sensitivity (94%) and specificity (92%) to detect ACM^8,9^.

In the current study cohort, APWA differentiated three distinct groups of patients with no (ACM-stage 1), moderate (ACM-stage 2) or extensive ACM (ACM-stage 3). Importantly, these patients were comparable regarding common cardiovascular risk factors such as age, hypertension, heart failure, diabetes or CHA2DS2-VASC-score.

### Atrial contractility, thrombogenesis and adverse cardiovascular outcomes in ACM

The loss of atrial contractility in ACM was in recent years suggested as a leading risk factor promoting left atrial thrombogenesis and overt stroke in patients with AF^12^. LAA-flow velocities in the current study were inversely related to ACM-severity and decreased continuously from patients with ACM-stages 1 (no ACM) to 3 (extensive ACM). Of note, up to 68% of patients in ACM-stage 2 and 3 had LAA-flow velocities ≤ 20 cm/s, which was previously shown as an independent risk factor for overt stroke^12^. In line with these findings, almost all patients with LAA-Thrombus (97% [106/109]) had electrocardiographic evidence of at least moderate ACM, supporting a pathophysiological relationship of ACM, reduced atrial contractility, and increased risk for atrial thrombus formation.

Extensive ACM (ACM-stage 3) in the current study in addition was associated with an increased rate of adverse cardiovascular events, which was driven primarily by all-cause death and hospitalization for heart failure. While the mechanisms behind this association are beyond the scope of the current study, screening for extensive ACM may be useful to identify patients with an overall elevated risk for cardiovascular events.

These findings are of particular importance as propensity-score matching in the current study resulted in patient cohorts with and without LAA-thrombus that were comparable with regard to established cardiovascular comorbidities. The aforementioned discriminating stages of ACM were therefore identified in a study population that would otherwise be considered homogeneous using conventional risk stratification models such as the CHA2DS2-VASC-score.

### Limitations

In the current study, propensity-score matching resulted in an evenly distributed patient group with regard to the most important cardiovascular risk factors such as age, hypertension and diabetes, heart failure, relevant medications such as anticoagulants and antithrombotics, and clinical phenotype of AF. Each of these factors was previously individually or in combination shown to relate to left atrial thrombus formation or stroke^3,19^. While APWA successfully identified patients with ACM and LAA-thrombus in this homogeneous patient group with regard to traditional cardiovascular risk factors, the matched nature of our study cohort does not allow to derive a proper risk stratification model such as CHA2DS2VASC-score. The study encompasses exclusively patients that were referred for cardioversion, which may represent a certain selection bias.

Also, ischemic stroke and LAA thrombus-formation as the pre-clinical event are two separate entities, and the current study was not powered to prospectively investigate the likelihood of ischemic stroke in the presence or absence of ACM. A major impact of reduced LAA contractility/flow and presence of LAA thrombus on future risk of ischemic stroke, however, was demonstrated in numerous trials^12^. It is therefore highly probable that our findings are transferable to the clinical event of ischemic stroke as well, though future confirmative studies are warranted. As the majority of patients in the current study had LAA-thrombus despite oral anticoagulation, the generalization of the reported findings to OAC-naive patients remains to be validated. However, the distribution of ACM-stages in OAC-naive patients did not differ from patients using OAC (Supplemental Fig. 6). Also, the currently proposed algorithm for ACM-staging using P-wave duration and –morphology requires an ECG with at least a few sinus beats recorded, making it inapplicable to the subgroup of patients in whom such sinus rhythm-ECG is unavailable for any reason.

## Conclusion

The current study demonstrates that ACM-severity as assessed by APWA relates to left atrial contractile dysfunction, thrombogenesis, and adverse cardiovascular outcomes including death. Diagnosis of ACM using APWA might provide a promising approach to improve future risk stratification models in primary prevention of stroke and other cardiovascular diseases.

## Supplementary Information

Supplementary Information.
