# Supplementary material for: Electrocardiographic diagnosis of atrial cardiomyopathy to predict atrial contractile dysfunction, thrombogenesis and adverse cardiovascular outcomes
Source: Sci Rep. 2022 Jan 12;12:576. doi: 10.1038/s41598-021-04535-7 (PMC8755780; doi:10.1038/s41598-021-04535-7)
Supplement: Supplementary file 1 — Supplementary Information. [file 41598_2021_4535_MOESM1_ESM.pdf]

Electrocardiographic Diagnosis of Atrial Cardiomyopathy to Predict Atrial Contractile Dysfunction, Thrombogenesis and Adverse Cardiovascular Outcomes

-Supplementary Materials-

Figure Legends

Supplemental Figure 1: Propensity-Score Matching

Histograms of the density of propensity scores for observations before and after matching with overlaid kernel density estimate (A), dotplots of the propensity scores before and after matching (B), and the dotplots of the standardized mean differences for all covariates before and after matching (C) are given.

Supplemental Figure 2: Study outline

LAA, left atrial appendage; TEE, transesophageal echocardiography

Supplemental Figure 3:

Patients with impaired flow ≤20cm/s (in red) in the LAA demonstrate prolonged amplified P-wave APW-duration. The dotted line represents the diagnostic criterion for fibrotic atrial myopathy (APW-duration ≥150 ms, **B)**. Patients with impaired LAA-flow ≤20cm/s (in red) more often demonstrate an interatrial block ECG-pattern (biphasic p-waves in inferior leads II, III and aVF) and/or late-terminal-P ECG pattern (late positive deflection in lateral leads I, aVL, V2-6, **B)**.

APW, amplified p-wave; LAA, left atrial appendage

Supplemental Figure 4:

APW-duration in patients with LAA-thrombus (in red) at transesophageal echocardiography is significantly prolonged as compared to propensity-score matched control patients without LAA thrombus (in blue, **A)**. The percentage of patients with specific pathological ECG-patterns (interatrial block (IAB) and late-terminal P) with regard to the presence or absence of LAA-Thrombus are shown in panel **(B)**.

Supplemental Figure 5:

Kaplan-Meier-curves for survival free of MACCE (stroke or transient ischemic attack, myocardial infarction, hospitalization for heart failure or all-cause death) in patients with ACM-stages 1 (no ACM, in green), 2 (moderate ACM, in orange) or 3 (extensive ACM, in red), adjusted for age, sex, diabetes, vascular disease, hypertension and left-ventricular dysfunction.

Supplemental Figure 6:

Overview of ACM-stages (ACM-stage 1 in green, ACM-stage 2 in orange, ACM-stage 3 in red) in patients with LAA-thrombus and matched control patients. Left, patients without prior oral anticoagulants, right patients under oral anticoagulants.

APWA, amplified P-wave analysis; ACM, atrial cardiomyopathy

Supplemental Figure 1

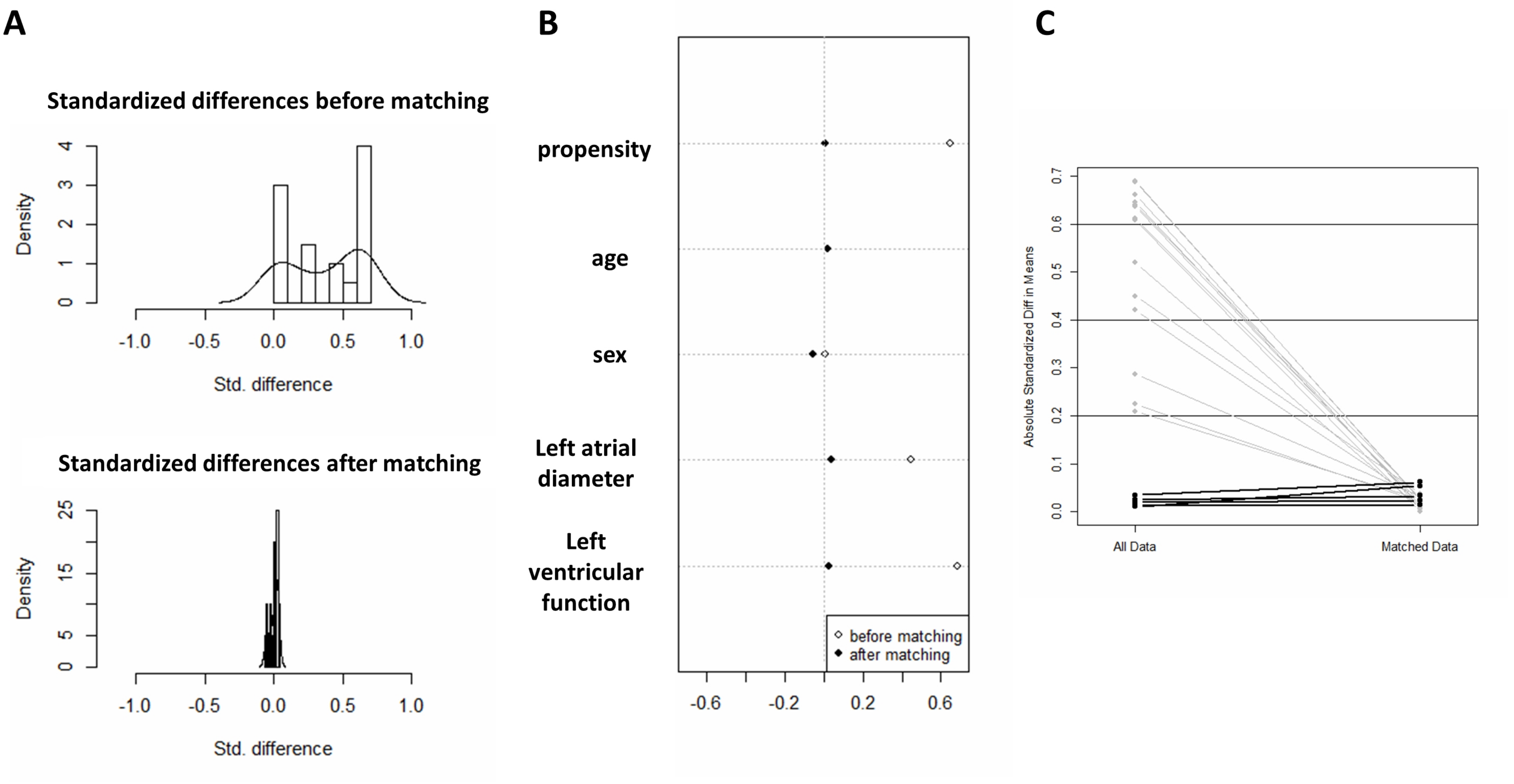

Supplemental Figure 2

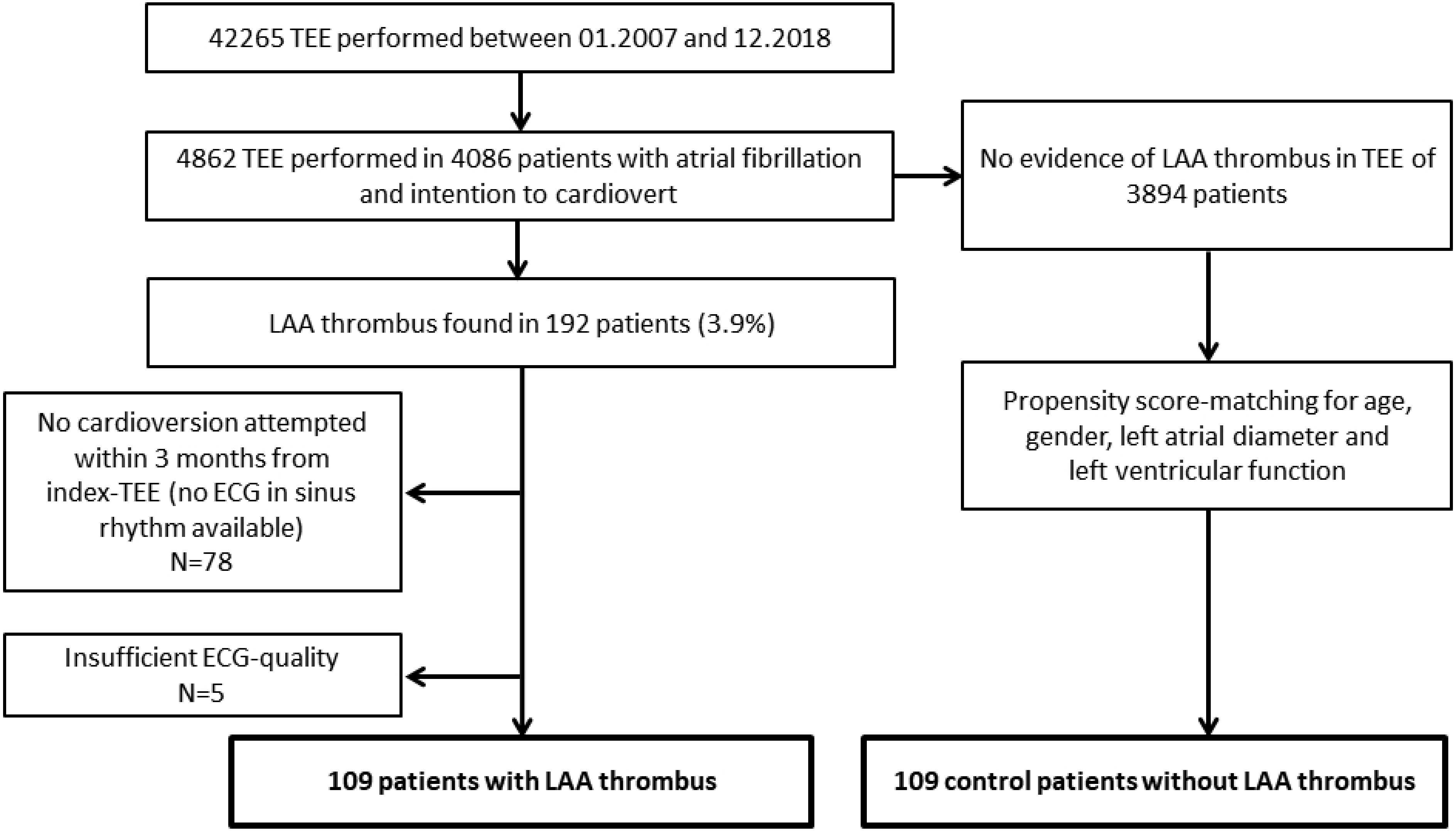

SUPPLEMENTAL FIGURE 3

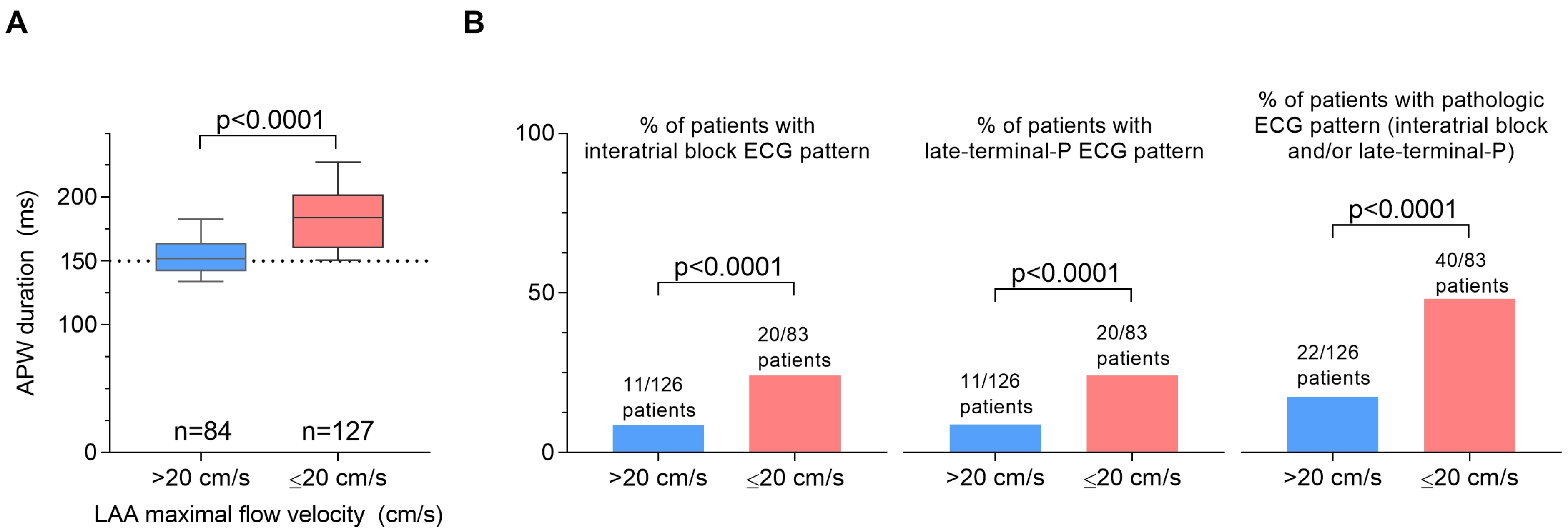

SUPPLEMENTAL FIGURE 4

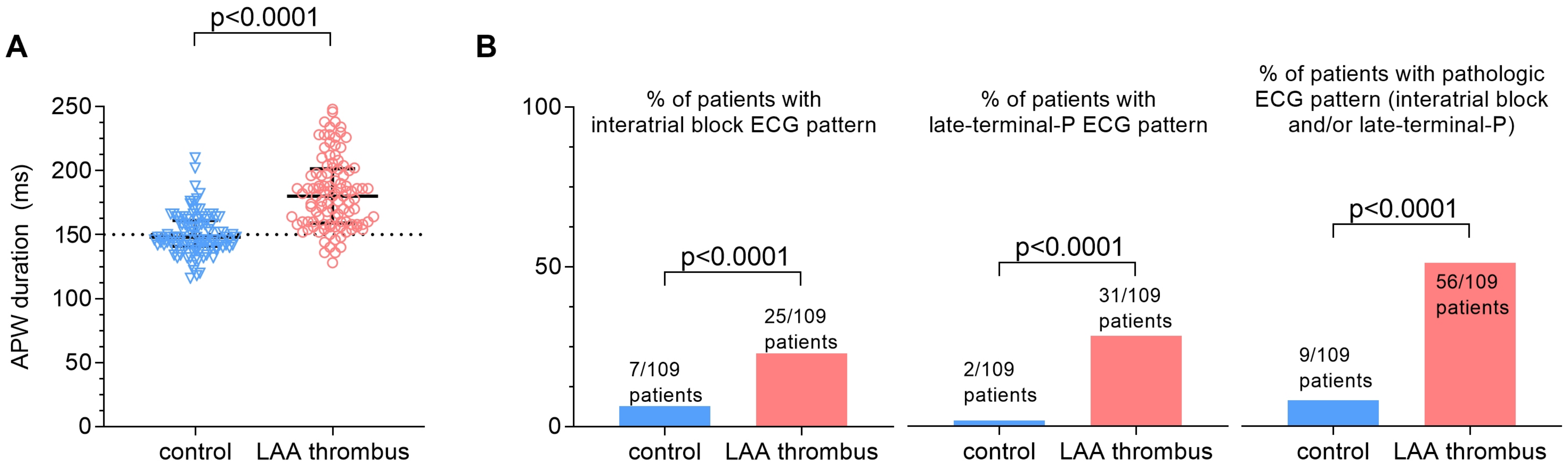

Supplemental Figure 5

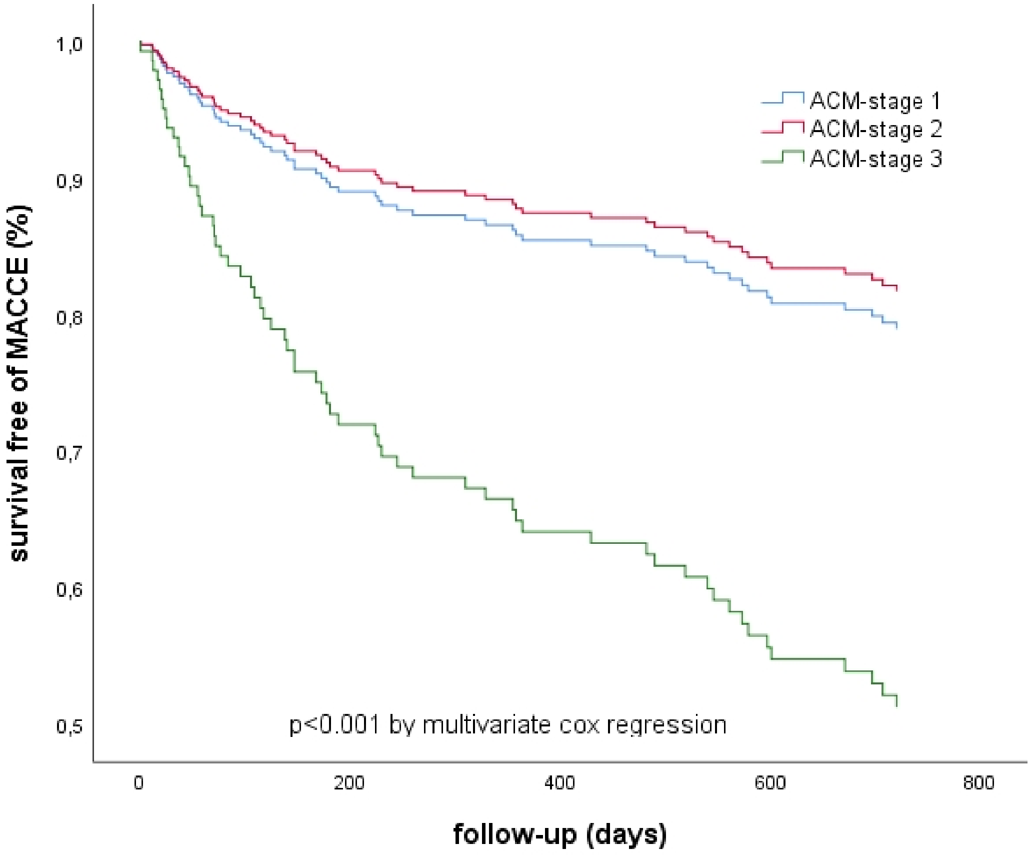

Supplemental Figure 6

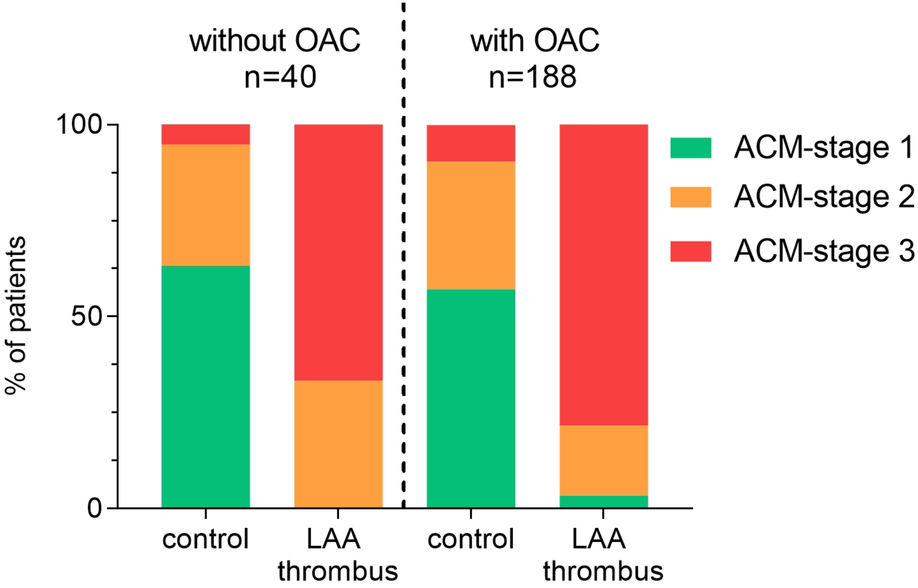

Supplemental Table 1: Logistic regression analyses of predictors for left-atrial contractile dysfunction

	Multivariate analyses		
**variable**	**OR**	**95% CI**	**P value**
age	1.008	0.96-1.06	0.345
male sex	0.881	0.29-2.68	0.823
diabetes	1.067	0.30-3.76	0.920
LV systolic dysfunction	4.046	1.23-13.29	0.021
history of stroke or TIA	1.109	0.23-5.28	0.897
vascular disease	0.480	0.16-1.42	0.187
hypertension	1.294	0.38-4.41	0.681
prior oral anticoagulants	1.124	0.32-3.99	0.857
APWA-derived ACM-stage (per increase in stage)	6.841	3.40-13.75	<0.001

ACM, atrial cardiomyopathy; APWA, amplified p-wave analysis; LV, left ventricular; TIA, transient ischemic attack
